# Domestic travel as a driver for the dissemination of *mcr-1* in healthy travelers in China: a prospective, genomic epidemiological and gut microbiome study

**DOI:** 10.1128/aac.01746-25

**Published:** 2026-04-20

**Authors:** Meina Wu, Yushun Chen, Minxuan Su, Hao Wu, Li Luo, Xiaojun Ao, Chanjing Zhao, Jingwei Shui, Shuan Wen, Jiehao Lin, Jieying Pu, Jianming Zeng, Yueting Jiang, Hoiman Ng, Zhongde Zhang, Mingli Hu, Bin Huang, Lingqing Xu, Cha Chen, Cong Shen

**Affiliations:** 1 Clinical Laboratory/State Key Laboratory of Traditional Chinese Medicine Syndrome, Guangzhou University of Chinese Medicine, The Second Clinical Medical College, Guangdong Provincial Hospital of Chinese Medicine, Guangzhou, China; 2 State Key Laboratory of Dampness Syndrome of Chinese Medicine, Guangdong Provincial Hospital of Traditional Chinese Medicine 600607, Guangzhou, China; 3 Guangdong Provincial Key Laboratory of Research on Emergency in TCM, Guangzhou, China; 4 Department of Otolaryngology, Hospital of Honghe State Affiliated to Kunming Medical University, Southern Central Hospital of Yunnan Province 735006 , Mengzi, China; 5 Clinical Laboratory, First Affiliated Hospital of Guangzhou Medical University 117969, Guangzhou, China; 6 Clinical Laboratory, Kiang Wu Hospital 105130, Macau, China; 7 Guangzhou University of Chinese Medicine, Science and Technology Innovation Center 47879, Guangzhou, China; 8 Clinical Laboratory, The First Affiliated Hospital of Sun Yat-sen University 71068 , Guangzhou, China; 9 The Sixth Affiliated Hospital of Guangzhou Medical University, Qingyuan People's Hospital 534795, Qingyuan, China; Universita degli studi di Roma La Sapienza, Rome, Italy

**Keywords:** *mcr-1*, plasmid, domestic travel, colistin resistance, *Escherichia coli*, microbiome

## Abstract

The global spread of plasmid-mediated colistin resistance gene *mcr-1* poses a significant threat to public health. Although international travel is a known driver of antimicrobial resistance, the role of domestic travel in high-prevalence settings remains unclear. We conducted a prospective cohort study of 81 healthy volunteers traveling in China (June–September 2022). Fecal samples collected before and after travel were screened for *mcr-1*-positive *Escherichia coli* (MCRPEC). Antimicrobial resistance genes (ARGs), virulence factors (VFs), plasmid replicons, and gut microbial dynamics were investigated using whole-genome sequencing and 16S rRNA sequencing. Risk factors were analyzed using logistic regression analysis. Of the 81 participants who were negative for *mcr-1* at baseline, 12 (14.8%) acquired *mcr-1* after travel. Acquisition was associated with residence near poultry farms (odds ratio [OR] = 5.9, *P* = 0.04) and diarrhea during travel (OR = 11.22, *P* = 0.027). MCRPEC exhibited marked genetic diversity comprising 10 sequence types and the carriage of additional 23 ARGs and nine adherence-associated VFs. *mcr-1* was located on IncI2, IncX4, IncHI2, or IncP plasmids, with 91.7% (*n* = 11) transferable in conjugation assays. Gut microbiome analysis showed increased *α*-diversity, but a stable community structure, indicating colonization without major disruption. Our study demonstrated that domestic travel in China substantially contributes to the dissemination of *mcr-1*. Poultry exposure and gastrointestinal disturbances are key risk factors. Genetic diversity, plasmid transferability, and co-carriage of resistance and virulence determinants highlight the risk of onward spread. Antimicrobial resistance surveillance should extend beyond international travel and incorporate domestic mobility within a “One Health” framework.

## INTRODUCTION

Colistin is considered a last-line antimicrobial for the treatment of multidrug-resistant gram-negative bacterial infections, particularly those caused by carbapenem-resistant Enterobacteriaceae (CRE) (1, 2). However, the emergence and worldwide spread of plasmid-mediated colistin resistance, primarily mediated by *mcr-1*, critically undermine the effectiveness of one of the few remaining therapeutic options for treating difficult infections (3, 4). The *mcr-1* gene is primarily located on plasmids and within the transposon Tn*6330*, which enhances the potential for horizontal gene transfer (HGT) and facilitates its spread across diverse bacterial hosts, resulting in global prevalence and dissemination of *mcr-1* (5, 6).

International travel has been recognized as an essential driver of antimicrobial resistance (AMR) dissemination (7–11). Numerous studies have documented the high prevalence and frequent acquisition of extended-spectrum *β*-lactamases (ESBLs) producing Enterobacteriaceae and other significant pathogens among international travelers (9, 12). Travelers exposed to novel microbial environments have an increased probability of acquiring resistant bacteria, which could subsequently be introduced into their home communities upon return (8, 10, 13). Recent research has revealed that a notable proportion of travelers returning from regions with a high prevalence of *mcr-1* are carriers of *mcr-1*-positive Enterobacterales, highlighting the potential risk associated with traveling to areas with a high prevalence of antimicrobial resistance genes (ARGs) (10, 11, 14, 15).

Growing evidence indicates the spread of AMR linked to international travel. However, surveillance systems and population-based data on AMR acquisition during domestic travel in China are limited, leaving the role of domestic travel in AMR transmission dynamics unclear, particularly in regions with a high prevalence of *mcr-1* (7, 8). The initial dissemination of *mcr-1* was associated with animal reservoirs in China, and a subsequent decline in its prevalence was noted following the prohibition of colistin as a feed additive (16, 17). However, the persistent detection of *mcr-1* in human populations across various regions suggests that other factors, such as population mobility, may sustain residual circulation.

We conducted a longitudinal study to investigate the acquisition of *mcr-1* and its related risk factors in healthy volunteers who traveled within China. Furthermore, we characterized the genomic features of *mcr-1*-positive *Escherichia coli* (MCRPEC) and analyzed gut microbiome dynamics in travelers who acquired MCRPEC. Our findings highlight the role of domestic travel in the dissemination of *mcr-1* and emphasize the need to consider human mobility in AMR surveillance and control strategies.

## MATERIALS AND METHODS

### Study design and population

A prospective longitudinal cohort study was conducted on healthy undergraduate students planning to travel within China during the summer vacation period (from June to September in 2022). The inclusion criteria for volunteers were no history of cardiopulmonary disease, metabolic syndrome, dyslipidemia, gastrointestinal disease, and no use of antimicrobials within the 3 months before the first sample collection. All participants completed the study with no loss to follow-up.

### Sample collection

Volunteers provided fecal samples within 3 days before departure and within 3 days after returning from travel. Fecal samples were collected using a sterilized tube and transported to the laboratory within 1 h, where they were stored at −80°C until further processing. All volunteers completed a questionnaire after their return, which collected personal information, including age and gender, dietary habits, hospitalizations, antimicrobial use, living in proximity to poultry farms (<1 km) and other relevant factors (Table S1).

### Purification and identification of MCRPEC

MCRPEC was identified according to our previous studies (16). Samples were inoculated into 3 mL of brain heart infusion (BHI) broth (Oxoid, UK) supplemented with 2 mg/L colistin. The cultures were then incubated at 37°C for 18–24 h. Total DNA was extracted from 1 mL of the broth by the boiling method and screened for the presence of *mcr-1* to *mcr-5* and *mcr-7* to *mcr-9* by polymerase chain reaction (PCR), as previously described (16, 18). *mcr-*Positive samples were then cultured on MacConkey agar supplemented with 2 mg/L colistin. Subsequently, colonies with distinct morphologies were all selected for species identification. When fewer than 10 morphological types were present, additional colonies were chosen to ensure that at least 10 colonies from each sample were examined. Species identification was conducted using matrix-assisted laser desorption/ionization time-of-flight mass spectrometry (MALDI-TOF MS, VITEK MS, bioMérieux, France).

### Antimicrobial susceptibility testing

The minimum inhibitory concentrations (MICs) of the 12 antimicrobials were measured using the agar dilution method, whereas colistin and tigecycline were determined using the broth dilution method. The breakpoints used to define antimicrobial resistance were selected according to the Clinical and Laboratory Standards Institute (CLSI, M100-S34). The European Committee on Antimicrobial Susceptibility Testing (EUCAST) clinical breakpoints v15.0 were selected for tigecycline. *E. coli* ATCC 25922 was used as a quality control strain.

### Plasmid conjugation assay

A streptomycin-resistant *E. coli* C600 was used as the recipient strain. Donor and recipient isolates were cultured overnight and subcultured at a 1:100 ratio for 4 h at 37°C. The donor and recipient were then mixed at a 1:9 ratio, incubated stationary for 6 h at 37°C, and plated on Luria-Bertani (LB) agar plates supplemented with streptomycin (2,000 mg/L) and colistin (2 mg/L). Colonies were subjected to PCR and Sanger sequencing to detect the *mcr-1* gene and ST. Colonies positive for *mcr-1*, which also exhibited the same ST as the recipient strains, were identified as transconjugants (16).

### Whole-genome sequencing and bioinformatic analysis

The genomic DNA of MCRPEC was extracted and sequenced using the Illumina HiSeq 4000 platform. Raw reads were assembled using SPAdes v3.14.0 with "--careful" parameter (19). *In silico* multilocus sequence typing (MLST) was identified by mlst v2.16.2 against PubMLST database (https://pubmlst.org/). The antimicrobial resistance genes (ARGs), virulence factors (VFs), and plasmid replicons were identified by ABRicate v1.0.1  (https://github.com/tseemann/abricate) against the Comprehensive Antimicrobial Resistance Database (CARD), Virulence Factor Database (VFDB), and PlasmidFinder Database, respectively. Core-genome SNPs were identified using Snippy with the *E. coli* K-12 substr. MG1655 (GCA_000005845.2). A phylogenetic tree based on cgSNPs was generated using RAxML-ng under the general time-reversible + gamma rate heterogeneity model (GTR + G) and subsequently visualized via iTOL (https://itol.embl.de/) (20).

The structure of *mcr-1*-harboring plasmids was reconstructed according to our previous study (21). Briefly, the contigs were mapped against reference plasmid sequences using BLASTn analysis, including IncI2 (pHNSHP45, GenBank accession: KP347127.1), IncX4 (pIBMC_*mcr1*, MF449287), IncHI2 (pHNSHP45-2, KU341381.1), and IncP (pHNEP124, MT667260.1). The contigs that met the criteria (coverage > 60% and identity > 90%) were considered as candidate sequences, which were subsequently aligned and ordered according to the corresponding reference plasmid. The gaps between contigs were filled by mapping of raw reads to the reference plasmid sequence using BWA and BCFtools (22, 23). The reconstructed sequences were compared by BLASTn and visualized using Blast Ring Image Generator (BRIG). The genome assemblies of MCRPEC reported in this study have been deposited in the National Center for Biotechnology Information (NCBI) GenBank genomic DNA database under BioProject accession number PRJNA593695.

### Gut microbiome analysis

DNA was extracted from fecal samples using a QIAamp PowerFecal DNA Kit (QIAGEN, Hilden, Germany). The hypervariable V3 and V4 regions of the 16S rRNA gene were amplified and sequenced by Illumina MiSeq platform. Raw read processing and amplicon sequence variant (ASV) clustering were analyzed using QIIME2 v2021.11 (24). Taxonomic assignment of ASVs was performed on the SILVA 138 database. Shannon index and Bray-Curtis distance were estimated using the vegan package (https://cran.r-project.org/web/packages/vegan/index.html). The linear discriminant analysis effect size (LEfSe) method was implemented to identify differences in bacterial communities using the microeco package.

### Statistical analysis

Univariate logistic regression was used to determine the factors associated with MCRPEC colonization by SPSS software v27.0. Factors with a *P* < 0.2 were selected to generate the final parsimony model. Odds ratios (ORs) and the corresponding 95% confidence intervals (*CIs*) were calculated. A *P* value less than 0.05 was considered statistically significant.

## RESULTS

### Acquisition of *mcr-1* and associated risk factors

**Fig 1:**
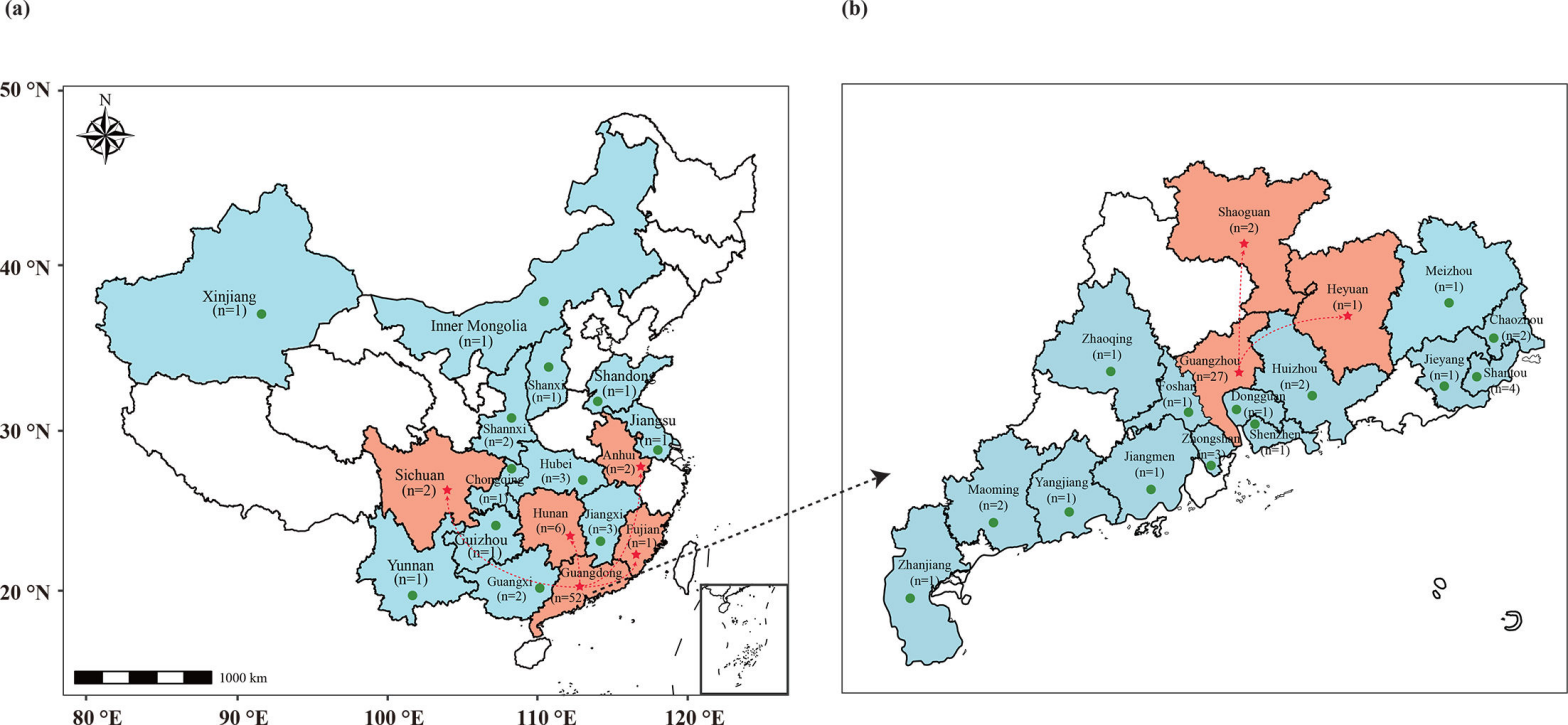
Geographic locations of travel destinations in (**a**) 16 provinces and (**b**) Guangdong province among 81 volunteers. Green circles denote destinations visited by volunteers, while red stars indicate destinations with volunteers who were positive for *mcr-1* in their post-travel fecal samples. The red dashed lines represent travel routes.

Of the 89 participants in the study, individuals with pre-travel samples positive for *mcr* genes were excluded, including eight individuals carrying the *mcr-1*, of whom two also carried the *mcr-9*. The median age of the remaining 81 participants was 21 years (interquartile range [IQR]: 19–24), with 67.9% (*n* = 55) being female (Table S2). The participants' travel destinations displayed considerable geographic diversity, including seven individuals traveling within Guangdong Province and five visiting other provinces across China, including Anhui, Fujian, Hunan, and Sichuan (Fig. 1). Of the 81 volunteers confirmed negative for *mcr* variants before travel, 14.8% (*n* = 12) of them were found to be positive for the *mcr-1* gene in their post-travel fecal samples.

Logistic regression analysis revealed two key risk factors significantly associated with acquiring *mcr-1*, including living near poultry farms (OR = 5.90, 95% CI: 1.08–32.15, *P*=0.04) and having diarrhea during travel (OR = 11.22, 95% CI: 1.31–95.95, *P* = 0.027) (Table 1). However, self-reported antimicrobial use and healthcare facility visits during travel were not significantly associated with the acquisition of *mcr-1*, potentially due to the limited sample size, which reduced statistical power.

**TABLE 1:** Risk factors linked to acquiring *mcr-1* during travel^*a*^

		*mcr-1*-Negative (*n* = 69)	*mcr-1*-Positive (*n* = 12)	Unadjusted *P* value	Adjusted *P* value	*OR* (95% *CI*)
Gender	Male	22 (31.88%)	4 (33.33%)	0.921	–	–
	Female	47 (68.12%)	8 (66.66%)			
	<18.5	8 (11.59%)	1 (8.33%)	0.815	–	–
BMI (kg/m²)	18.5 ≤ BMI ≤ 24.9	51 (73.91%)	11 (91.67%)			
	≥25.0	10 (14.49%)	0 (0.00%)			
Diarrhea		8 (11.59%)	5 (41.67%)	0.015	0.027	11.222 (1.312–95.954)
Visiting health care facilities		22 (31.88%)	3 (25.00%)	0.635	–	–
Living in proximity to poultry farms		7 (10.14%)	3 (25.00%)	0.163	0.040	5.898 (1.082–32.153)
Living with healthcare workers		17 (24.64%)	3 (25.00%)	0.979	–	–
Swimming		19 (27.54%)	5 (41.67%)	0.328	–	–
Contacting with pets		15 (21.74%)	1 (8.33%)	0.303	–	–
Antibiotic use		3 (4.35%)	1 (8.33%)	0.563	–	–
Gastrointestinal medications use		10 (14.49%)	4 (33.33%)	0.123	0.713	0.672 (0.081–5.578)
Eating undercooked or raw food		33 (47.83%)	6 (50.00%)	0.889	–	–

^*a*^ Data are represented as *n* (%). Unadjusted *P* value: univariate tests. All those testing at *P* < 0.2 were selected for multivariable analysis. Adjusted *P* value: multivariable model includes diarrhea, living in proximity to poultry farms, gastrointestinal medications use. –, variables not included in the multivariable model, with no estimated odds ratio or *P* value.

### Species and antimicrobial susceptibility profiles of *mcr-1*-positive isolates

Through the purification and identification of *mcr-1*-positive isolates, we identified all isolates from 12 volunteers as *E. coli*. All 12 MCRPEC isolates were multidrug-resistant, displaying a broad range of resistance patterns to various antimicrobial classes. All of the isolates were resistant to ampicillin and gentamicin, followed by ciprofloxacin (75.0%, *n* = 9), amikacin (33.3%, *n* = 4), fosfomycin (16.7%, *n* = 2), and cefotaxime (16.7%, *n* = 2). Conversely, ceftazidime resistance was detected in only a single isolate (8.3%). All 12 isolates remained susceptible to the critical last-resort antimicrobials, tigecycline, imipenem, meropenem, and nitrofurantoin (Table 2; Table S3).

**TABLE 2:** Antimicrobial resistance of MCRPEC isolates^*a*^

Categories of antibiotics	Antimicrobial agent	R (%)
Polymyxins	CST	12 (100.00%)
Tetracycline	TGC	0 (0.00%)
β-lactam	AMP	12 (100.00%)
	CTX	2 (16.67%)
	CAZ	1 (8.33%)
Carbapenems	IPM	0 (0.00%)
	MEM	0 (0.00%)
Fosfomycin	FOS	2 (16.67%)
Nitrofurans	NIT	0 (0.00%)
Aminoglycosides	AMK	4 (33.33%)
	GEN	12 (100.00%)
Quinolones	CIP	9 (75.00%)

^*a*^ CST, colistin; TGC, tigecycline; AMP, aminopenicillin; CTX, cefotaxime; CAZ, ceftazidime; IPM, imipenem; MEM, meropenem; FOS, fosfomycin; NIT, nitrofurantoin; AMK, amikacin; GEN, gentamicin; CIP, ciprofloxacin. The numbers in the table refer to the percentages of isolates showing resistance to each antimicrobial, which are represented as *n* (%).

### Genomic characterizations of MCRPEC isolates

**Fig 2:**
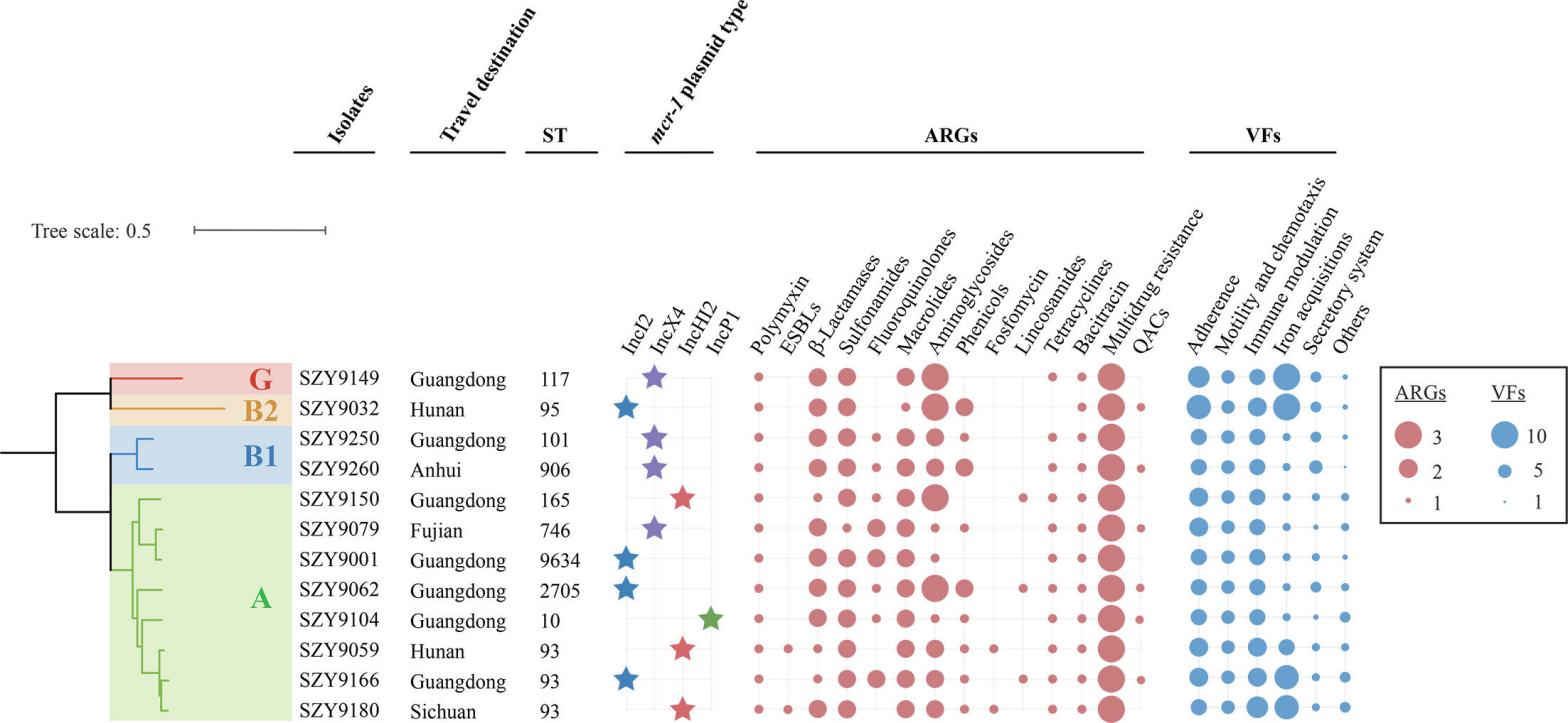
Phylogenetic tree and genomic characterizations of 12 MCRPEC isolates. The phylogenetic tree is located to the left of the plot, with different colors representing distinct phylogroups: red, yellow, blue, and green, corresponding to phylogenetic groups G, B2, B1, and A, respectively. The first column represents the strain names. The second column indicates the travel destinations. The third column represents ST. Blue, purple, red, and green stars represent the *mcr-1* plasmids IncI2, IncX4, IncHI2, and IncP, respectively. Red and blue circles illustrate the distributions of the functions related to the ARGs and VFs for each strain, with circle size proportional to the number of genes present in this category.

WGS analysis of 12 MCRPEC isolates revealed a highly diverse genetic landscape. Ten distinct STs were identified among the 12 isolates, including ST93 (*n* = 3) and single isolates of ST10, ST95, ST101, ST117, ST165, ST746, ST906, ST2705, and ST9634. The sporadic distribution of these isolates in the phylogenetic tree further indicated significant genetic diversity of the host bacteria carrying the *mcr-1* gene, suggesting a polyclonal population rather than the dominance of a single epidemic clone. While the host backgrounds were diverse, the phylogroup distribution was more concentrated in phylogroups A and B1 (83.4%, *n* = 10), which are commonly associated with community-acquired or commensal strains, suggesting that the acquisition of MCRPEC likely occurred in non-hospital settings (Fig. 2) (10).

In addition to *mcr-1*, the MCRPEC isolates demonstrated extensive co-occurrence of an additional 23 ARGs spanning 12 antimicrobial classes (Table S4). Each isolate harbored a median of 13 ARGs (IQR: 12–14). All isolates possessed ARGs encoding resistance to sulfonamides (*sul* and *dfrA*-like), macrolides (*mph-* and *emr*-like), and aminoglycosides (*aph*-, *aac3*- and *ant*-like), as well as efflux pump genes associated with multidrug resistance (*acrAB-tolC* and *mdtABCD-tolC*), followed by phenicols (75%, *n* = 9, *floR* and *cmlA1*) and fluoroquinolones (66.7%, *n* = 8, *qnrS1* and *oqxAB*).

VFDB analysis revealed that the isolates harbored 42 (median = 27, IQR: 26–35) virulence-associated genes attributed to five categories of functions that were involved in adhesion (21.4%, *n* = 9), motility and chemotaxis (11.9%, *n* = 5), immune modulation (19.1%, *n* = 8), iron acquisition (23.8%, *n* = 10), secretory system (14.3%, *n* = 6), and others (9.5%, *n* = 4) (Table S5). All isolates harbored genes associated with adherence, including *csg*, *fim*, *htpB*, and *ilpA*, followed by *ecp* (91.7%, *n* = 11), *aslA* (83.3%, *n* = 10), *fdeC* (58.3%, *n* = 7), *pap* (25%, *n* = 3), and *sinH* (16.7%, *n* = 2). These findings indicate a high capacity for these isolates to breach mucosal barriers and maintain intestinal colonization. Genes associated with type III and II secretion systems were detected among the isolates, including *esp* (91.7%, *n* = 11) and members of the *gsp* family (66.7%, *n* = 8). In summary, these MCRPEC isolates demonstrated co-carriage of extensive ARGs and VFs, suggesting that their dissemination may also facilitate the co-dissemination of other ARGs and VFs.

### Diversity and transferability of *mcr-1*-harboring plasmids

**Fig 3:**
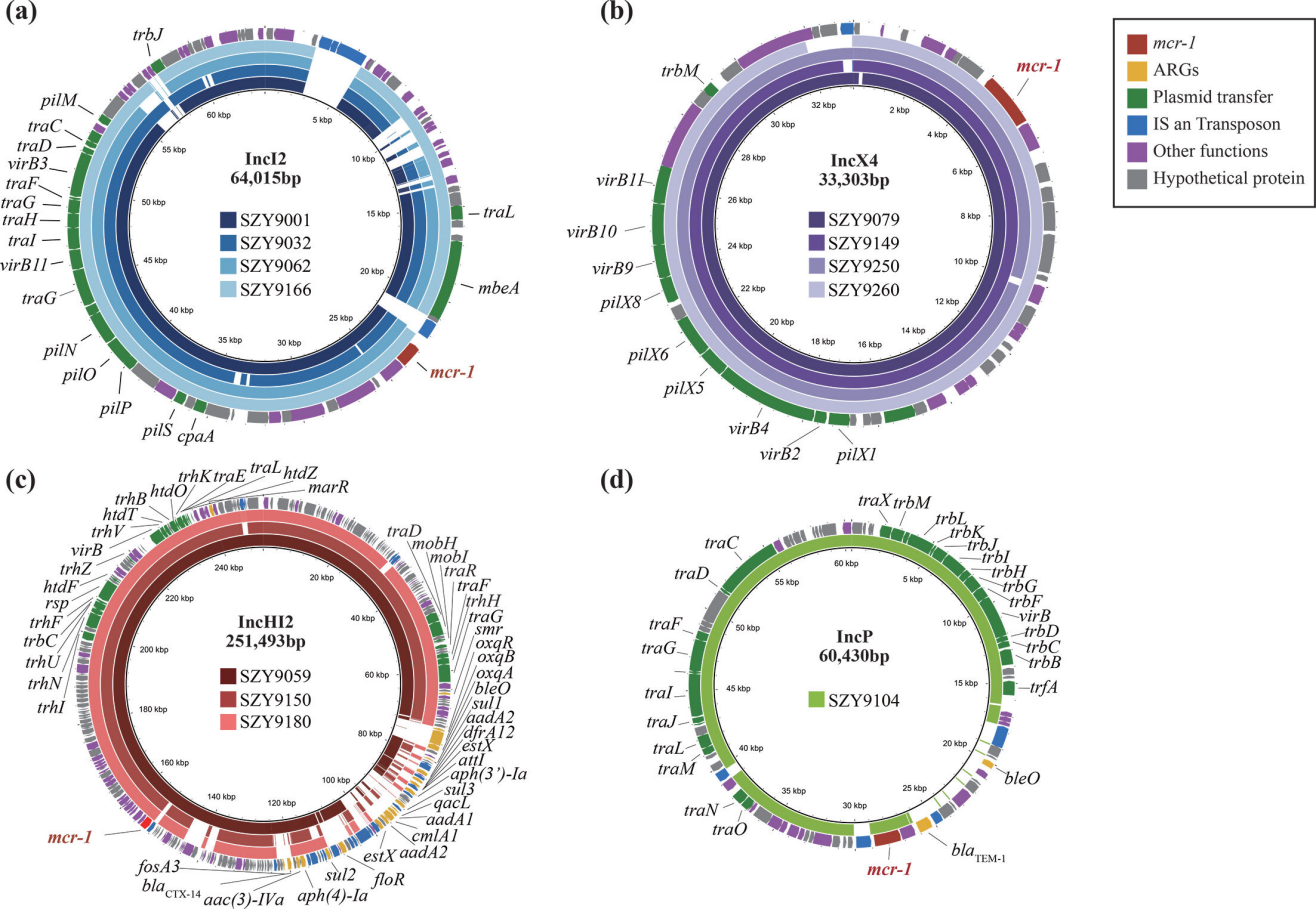
Structural comparisons of *mcr-1*-harboring plasmids. Comparisons of circular arrangements of *mcr-1*-harboring plasmids with reference genomes of (**a**) IncI2 (pHNSHP45, accession KP347127.1), (**b**) IncX4 (pIBMC_mcr1, accession MF449287), (**c**) IncHI2 (pHNSHP45-2, accession KU341381.1), and (**d**) IncP (pHNEP124, accession MT667260.1). The outer circles represent genome annotations with different colors for functions, while the inner circles show the plasmids used in this study.

The *mcr-1* gene identified in all 12 isolates was carried on four distinct plasmid incompatibility groups. IncI2 and IncX4 plasmids were the most prevalent, each representing 33.3% (*n* = 4) of the isolates, followed by IncHI2 (25.0%, *n* = 3) and IncP (8.3%, *n* = 1) (Fig. 3). Comparative plasmid analysis revealed that IncI2 and IncX4 plasmids exhibited a high degree of structural conservation, with only minor variations in insertion sequences (Fig. 3a and b). In contrast, IncHI2 plasmids displayed substantial structural heterogeneity, likely attributable to extensive multidrug resistance regions within plasmid backbones (Fig. 3c).

Conjugation experiments demonstrated that 91.7% (*n* = 11) of the isolates were capable of transferring *mcr-1*–harboring plasmids to recipient strains (Table S6). This transferability is likely driven by the presence of multiple plasmid-associated conjugation genes, highlighting the substantial potential for travelers to act as reservoirs and vectors for the dissemination of *mcr-1* to other bacterial hosts.

To further elucidate the genetic context of *mcr-1*, the flanking regions were analyzed and compared with the reference transposon Tn*6330* (GenBank accession no. LC424783.1). The majority of isolates (91.7%, *n* = 11) carried an *mcr-1-pap2* cassette lacking flanking IS*Apl1* elements, whereas only a single IncHI2 plasmid retained one upstream IS*Apl1*. Given the high frequency of conjugation observed and the absence of a complete composite Tn*6330* structure in most isolates, these findings suggest that plasmid-mediated horizontal transfer, rather than transposon-driven mobilization, is the predominant mechanism facilitating *mcr-1* dissemination during travel.

### Changes in the gut microbiome of volunteers who acquired MCRPEC

**Fig 4:**
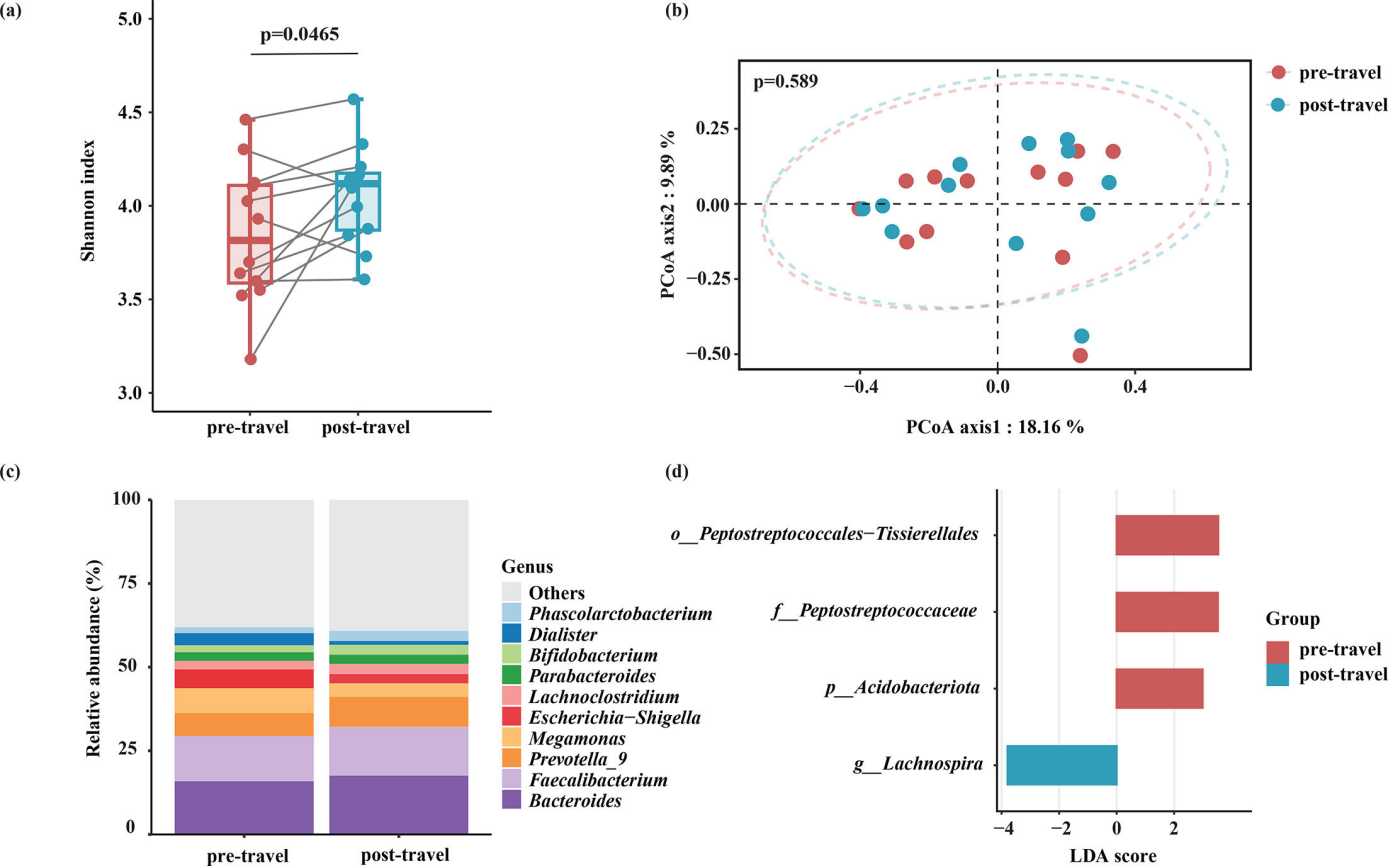
Gut microbiota dynamics in 12 volunteers who acquired MCRPEC. (**a**) Changes in Shannon index pre- and post-travel. (**b**) PCoA structure based on Bray-Curtis distance. *P* value was given by PERMANOVA. (**c**) Taxonomic compositions of the top 10 most abundant bacterial genera. (**d**) LDA score and the differentially abundant taxa identified by LEfSe.

A within-individual analysis of gut microbiota dynamics was conducted in the volunteers who acquired MCRPEC. The Shannon diversity index significantly increased in the gut microbial community following travel (*P* = 0.0465) (Fig. 4a). Despite this increase in *α*-diversity, no significant alterations were observed in the *β*-diversity of the overall gut microbiota structures during the travel period (*P* = 0.589) (Fig. 4b). The dominant and core genera, including *Bacteroides*, *Faecalibacterium*, and *Prevotella*, remained stable throughout the study (Fig. 4c). Differential abundance analysis using LEfSe revealed four taxa with significant changes in their relative abundance. The abundance of the genus *Lachnospira* increased markedly following travel (Fig. 4d), which may be related to changes in dietary fiber intake during the journey (25, 26). Conversely, a significant decrease was observed in the abundance of the phylum Acidobacteriota and family Peptostreptococcaceae (Fig. 4d). These observations collectively suggest that the successful acquisition of MCRPEC occurs, even when the overall stability of the gut microbiota is maintained, implying a high degree of adaptability and colonization potential of the newly acquired strains (9).

## DISCUSSION

This longitudinal investigation demonstrated that 14.8% of healthy individuals acquired the colistin resistance gene *mcr-1* during domestic travel in China, highlighting the significant role of human mobility in the dissemination and persistence of *mcr-1*, even in regions with restricted agricultural colistin usage (27). The proportion of travelers who acquired *mcr-1* in this study markedly exceeded the 1.5–4.9% reported among European international travelers, including those traveling to regions with high prevalence rates, such as Southeast Asia (9.2%) and India (11%) (10, 11, 13). This discrepancy is likely attributable to the endemic presence of *mcr-1* in these regions, potentially exacerbated by factors, such as travel conditions, food sanitation, and dietary habits, which differ from those typically encountered by travelers from other regions (28). While previous research has primarily shown that international travel contributes to the spread of antimicrobial resistance across regions with different prevalence levels, our results demonstrate that domestic travel within high-AMR areas is also a significant and frequently neglected risk factor, which emphasizes the urgent necessity to develop and enhance domestic AMR surveillance systems to complement current frameworks.

Despite the ban on colistin as a feed additive in China, we identified a high odds ratio for living near poultry farms, which suggests that environmental or animal reservoirs remain a significant and persistent source of human exposure. Farm exposure may contribute to the enrichment of ARGs within the gut microbiome, thereby collectively elevating the risk of MCRPEC colonization through environmental pathways (29). Our findings highlight a critical need for "One Health" approaches to AMR surveillance, integrating data from human populations with continuous monitoring of agricultural and environmental settings. As in previous studies, experiencing diarrhea while traveling raises the risk of acquiring *mcr-1* likely due to changes in diet and sanitation practices during travel (14, 30). This condition can temporarily compromise the integrity of the gut barrier, thereby facilitating colonization by pathogenic microorganisms, which has also been described in the acquisition of ESBL-producing Enterobacteriaceae among travelers. In contrast, the lack of a significant correlation with antimicrobial use should be interpreted with caution due to the small sample size of users (*n* = 4), which likely limits the statistical power to detect a true effect.

A diverse polyclonal population of MCRPEC isolates comprised 10 distinct sequence types, which is in contrast with our pre-ban findings of ST10 dominance, indicating that the dissemination of the *mcr-1* gene could not be attributed to the clonal expansion of a single successful clone (16, 17, 31). Interestingly, the predominance of community-associated phylogroups A and B1 aligns with acquisition patterns observed in international travel studies, suggesting common mechanisms, such as animal reservoirs and foodborne exposure (10). Notably, our observations indicate a high transferability of plasmids attributed to the presence of multiple genes associated with plasmid transfer functions, which suggests that the *mcr-1* gene could be transferred to the host’s native gut microbiota, even in cases where the strain does not establish long-term colonization (4, 6). These findings indicate that travelers act as vectors via two linked pathways: either helping diverse local MCRPEC isolates establish themselves or obtaining *mcr-1*-harboring plasmids that can spread to their native commensal *E. coli* populations. This dual role complicates surveillance and control strategies that need to address both resistant strains and the mobility of resistance genes.

*mcr-1* acquired by travelers was located on diverse plasmid types, which reinforces the conclusion that these travelers were exposed to multiple independent acquisition sources at their destinations (3). The predominant plasmid types are IncI2 and IncX4, both of which are known for their stability and efficient transmission (16, 31). Inconsistent with previous studies, we did not observe the composite Tn*6330* (IS*Apl1-mcr-1-pap2*-IS*Apl1*) in this study (16, 31). This loss may help stabilize the *mcr-1* cassette and likely reflects that dissemination of *mcr-1* is being driven primarily by plasmid transfer (5, 6). The high conjugation success rate observed in our study highlights the potential for onward transfer to other gut bacteria, even within a short timeframe of domestic travel, which suggests that travelers are effective vectors of plasmid-mediated resistance across regions, thereby complicating containment efforts (11, 15). Notably, the coexistence of a broad spectrum of ARGs, particularly those associated with *mcr-1*-harboring plasmids, may facilitate co-selection and dissemination of other clinically relevant determinants (5, 31). Although numerous ARGs were identified in the isolates, most strains remained phenotypically susceptible to commonly used antibiotics, which could be attributed to silent or poorly expressed ARGs that are not functionally active under standard conditions. Despite the observed susceptibility, these ARGs constitute a hidden reservoir that can be mobilized via horizontal gene transfer. Under antimicrobial selection pressure, such silent determinants may become activated or disseminated to other strains, potentially driving future resistance spread, which highlights the importance of integrating genomic and phenotypic data for comprehensive surveillance of antimicrobial resistance transmission risks. The extensive array of VFs, especially those involved in adhesion, iron acquisition, and motility, may enhance the capacity of these emerging strains to overcome ecological barriers and establish colonization within the host (31, 32).

Analysis of gut microbiome dynamics revealed that MCRPEC acquisition occurred without major disruption of the overall microbial community structure. Although Shannon diversity increased slightly after travel, the dominant genera remained stable, suggesting that MCRPEC can establish colonization within a resilient microbial ecosystem (33). This adaptability emphasizes the colonization potential of MCRPEC and raises concerns regarding the persistence of such strains in the community.

This study had several limitations. First, our sample size was relatively small, which may have limited the statistical power to detect other potential risk factors. Second, the study focused on domestic travel within China, and the findings may not be generalizable to other countries with different travel patterns and *mcr-1* epidemiology. Furthermore, the present study was unable to assess baseline AMR transmission within the community, as a control group of non-travelers was not included. Future investigations incorporating non-traveler cohorts would help to resolve it. Finally, the study design relies on a single post-travel sample, which does not provide information on the duration of carriage or the long-term impact on the host’s microbiome. Future research should focus on the duration and stability of *mcr-1* carriage following travel, the frequency of secondary transmission to close contacts, and the precise molecular mechanisms that enable the successful colonization of these multidrug-resistant strains in a stable gut environment, which are essential for developing effective strategies to mitigate the ongoing spread of this critical resistance gene.

Despite these limitations, our findings provide novel evidence that domestic travel within China plays a significant role in the spread of *mcr-1*. Additionally, we emphasize the complex interactions between environmental exposure, host factors, and microbial ecology that facilitate the acquisition of *mcr-1*. AMR surveillance programs should incorporate genomic surveillance, risk factor mitigation strategies, and microbiome-informed interventions to curtail the ongoing spread of *mcr-1*. Such programs should expand their scope beyond international travel to encompass domestic mobility, especially in regions characterized by intensive animal agriculture.

## Data Availability

The raw reads and assembled genomes of evolved populations and evolved clones reported in this study have been deposited in the NCBI GenBank database under BioProject accession numbers PRJNA1336721 and PRJNA1336720.

## SUPPLEMENTAL MATERIAL

Fig. S1 aac.01746-25-s0001.pdf ARGs and VFs of 12 MCRPEC isolates.

Table S1 aac.01746-25-s0002.xlsx Questionnaire used in this study.

Table S2 aac.01746-25-s0003.xlsx Basic information of 81 volunteers.

Table S3 aac.01746-25-s0004.xlsx MICs of 12 MCRPEC isolates for 12 antimicrobials.

Table S4 aac.01746-25-s0005.xlsx Distributions of ARGs for 12 MCRPEC isolates.

Table S5 aac.01746-25-s0006.xlsx Distributions of VFs for 12 MCRPEC isolates.

Table S6 aac.01746-25-s0007.xlsx Transconjugation ability of mcr-1-harboring plasmids from 12 MCRPEC isolates.

ASM does not own the copyrights to Supplemental Material that may be linked to, or accessed through, an article. The authors have granted ASM a non-exclusive, world-wide license to publish the Supplemental Material files. Please contact the corresponding author directly for reuse.
