# Supplementary figures and images for: Domestic travel as a driver for the dissemination of mcr-1 in healthy travelers in China: a prospective, genomic epidemiological and gut microbiome study
Source: Antimicrob Agents Chemother. 2026 Apr 20;70(6):e01746-25. doi: 10.1128/aac.01746-25 (PMC13231915; doi:10.1128/aac.01746-25)

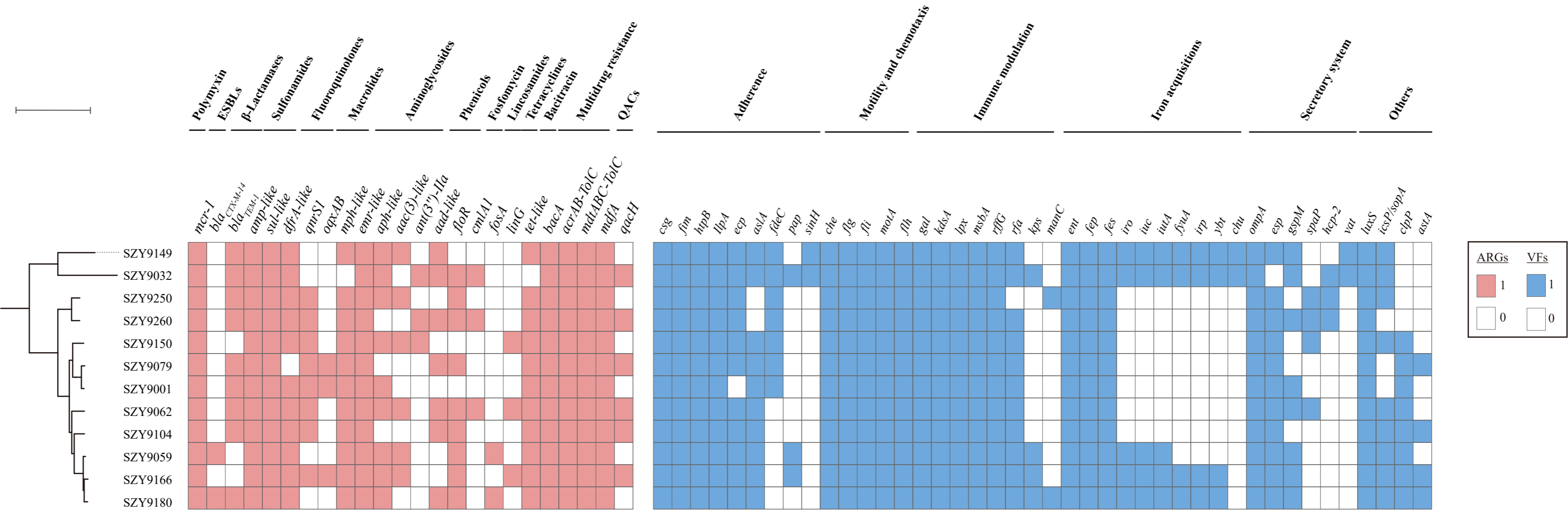

Supplementary Figure 1. Phylogenetic tree and genomic characterizations of 12 MCRPEC isolates.

Supplement: Fig. S1 — ARGs and VFs of 12 MCRPEC isolates. [file aac.01746-25-s0001.pdf]
